# 

**Published:** 1854-01

**Authors:** 


### CONTENTS.

| | Page | | Page |
|---|---|---|---|
| Editor's Address | 1 | Wearing the Beard | 15 |
| Subjects to be Treated | 5 | Invalids and Exertive Exercise | 16 |
| Eating and Drinking | 6 | Heart Disease | 17 |
| Tea and Coffee | 6 | Consumption and the South | 18 |
| Use of Milk | 7 | Book Notices | 22 |
| Dr. Nott's Munificence | 8 | "The Independent" | 22 |
| The food we Eat | 11 | The Home Journal | 22 |
| How to attain Old Age | 12 | The American Medical Monthly | 23 |
| Too white Flour | 13 | To Correspondents | 23 |
| Traveling for Health | 14 | A Premium | 24 |

